# Transfer Learning and Optimized Machine Learning Techniques for Multiclass Diabetic Retinopathy Classification Using Retinal Images

**DOI:** 10.3390/diagnostics16142189

**Published:** 2026-07-14

**Authors:** Mohammad Reza Yousefi, Ali Bakrani, Elias Ebrahimzadeh, Amin Dehghani

**Affiliations:** 1 Department of Electrical Engineering, Na.c., Islamic Azad University, Najafabad 8514143131, Iran; alibakrani73@gmail.com; 2 Digital Processing and Machine Vision Research Center, Na.c., Islamic Azad University, Najafabad 8514143131, Iran; 3 School of Electrical and Computer Engineering, College of Engineering, University of Tehran, Tehran 1439957131, Iran; e_ebrahimzadeh@ut.ac.ir; 4 School of Cognitive Sciences, Institute for Research in Fundamental Sciences (IPM), Tehran 1956836613, Iran

**Keywords:** diabetic retinopathy (DR), transfer learning (TL), deep learning, retinal imaging, convolutional neural networks (CNNs), machine learning (ML), medical image analysis

## Abstract

**Background/Objectives:** Diabetic Retinopathy (DR) is a prevalent and severe complication of diabetes, caused by prolonged hyperglycemia that damages retinal microvasculature and may ultimately lead to vision loss or blindness. While convolutional neural networks (CNNs) have shown promise in automating DR detection via retinal imaging, traditional approaches often suffer from limited diagnostic accuracy, long training times, and reliance on small or imbalanced datasets. **Objective:** This study evaluates an integrated transfer-learning using adaptive training strategies for multiclass retinal image classification. **Methods:** The proposed framework integrates transfer learning, feature-space dimensionality reduction, and adaptive training strategies based on an ImageNet pretrained ResNet50 backbone to improve training stability, computational efficiency, and multiclass retinal image classification performance. **Results:** The proposed Transfer Learning (TL)-based model was trained and evaluated on a large, publicly available dataset of retinal images, achieving an overall accuracy of 84%, maximum class-specific accuracy of 89%, sensitivity of up to 97%, and an F1-score of 92%. These results demonstrate reasonable overall classification performance under constrained data conditions. **Conclusions:** The proposed framework demonstrates the feasibility of integrating transfer learning and adaptive training strategies for multiclass retinal image classification under constrained benchmark conditions. However, the study is limited by the use of heavily downsampled retinal images, and further validation on high-resolution clinical datasets is required before practical deployment. Future methodological refinement and validation on high-resolution clinical datasets may support development of computer-assisted retinal image analysis systems.

## 1. Introduction

Early detection of diabetes is critically important for preventing long-term complications that can affect vital organs such as the kidneys, heart, blood vessels, and eyes [1,2,3,4,5,6,7,8,9,10]. Prompt diagnosis and effective management improve glycemic control, reduce the risk of comorbidities, and ultimately enhance patient outcomes. Among diabetes-related complications, diabetic retinopathy (DR) is one of the most common and vision-threatening conditions. It occurs due to chronic hyperglycemia, which damages the microvasculature of the retina, potentially leading to retinal hemorrhages, macular edema, and progressive vision loss [5,6,7,8,9].

Screening for DR using retinal imaging has proven to be a valuable approach for early intervention. By identifying pathological changes in the retina at early stages, healthcare providers can initiate timely treatments that help control blood sugar, prevent vision deterioration, and improve quality of life. Additionally, early diagnosis can reduce healthcare costs by avoiding the need for more intensive treatments during advanced stages of disease [1,2,3,4,5,9]. Since diabetes often progresses silently without noticeable symptoms, periodic retinal examinations are essential. Proper disease management may include medical and lifestyle interventions [11].

**Figure 1:**
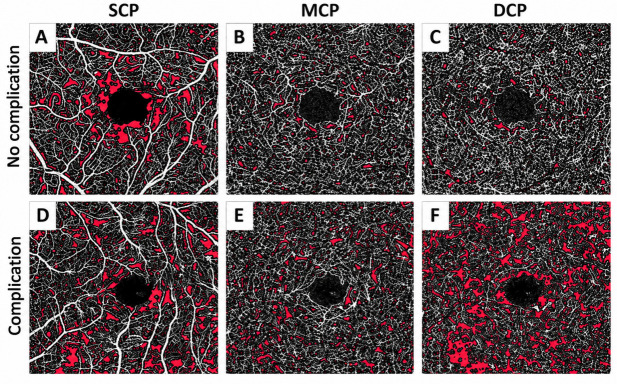
Representative optical coherence tomography angiography (OCTA) images of the superficial capillary plexus (SCP), middle capillary plexus (MCP), and deep capillary plexus (DCP). (**A**) SCP image from an eye without diabetic retinopathy-related complications. (**B**) MCP image from an eye without diabetic retinopathy-related complications. (**C**) DCP image from an eye without diabetic retinopathy-related complications. (**D**) SCP image from an eye with diabetic retinopathy-related complications. (**E**) MCP image from an eye with diabetic retinopathy-related complications. (**F**) DCP image from an eye with diabetic retinopathy-related complications. Red overlays indicate abnormal or non-perfused capillary regions, while the white lines represent the retinal vascular network. Adapted from Ong et al. [12].

Figure 1 visually demonstrates the differences between retinal images of individuals with DR and those without the condition, highlighting the importance of early identification of retinal damage.

**Figure 2:**
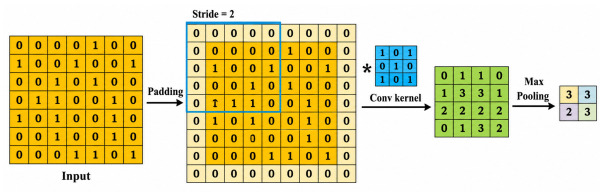
High-level schematic of a convolutional neural network (CNN) architecture used for image analysis. The processing pipeline consists of (1) an input image, (2) zero-padding to preserve spatial dimensions, (3) convolution using a 3 × 3 kernel filter, and (4) max-pooling to generate feature maps for image classification [24].

In recent years, artificial intelligence and machine learning have emerged as powerful tools for healthcare diagnosis and medical data analysis. These methods have been successfully applied across a range of clinical problems, including diabetes prediction, Alzheimer’s disease detection, brain tumor identification, COVID-19 detection from chest X-ray images, and biomedical signal analysis [13,14,15,16,17,18,19,20,21,22,23]. Building on these broader advances, retinal image analysis combined with machine learning (ML) has become a promising approach for automated diabetic retinopathy (DR) screening. Among machine learning techniques, convolutional neural networks (CNNs) are widely used for retinal image analysis because of their ability to learn hierarchical spatial features directly from images [24]. Figure 2 illustrates a high-level schematic of a CNN architecture for completeness.

While transfer-learning-based approaches have been widely used for diabetic retinopathy screening, most existing pipelines primarily focus on selecting a pretrained CNN architecture and fine-tuning it on retinal images. In contrast, the present study focuses on evaluating an integrated workflow that combines transfer learning, feature refinement, and adaptive training strategies within a unified diabetic retinopathy classification pipeline. The proposed approach systematically combines deep feature extraction from pretrained networks, feature-space dimensionality reduction, and adaptive training strategies within a single transfer-learning framework. This integrated workflow is designed to address common practical limitations of existing TL-based DR pipelines, including training instability, feature redundancy, and degraded performance in lower-severity NPDR stages.

Given the increasing global burden of diabetes and its direct impact on visual health, it is imperative to develop advanced tools for early detection of DR. The present study evaluates an integrated transfer-learning workflow that combines pretrained feature extraction, feature-space dimensionality reduction, and adaptive training strategies for multiclass diabetic retinopathy classification under constrained data conditions [25]. The framework utilizes an ImageNet pretrained ResNet50 backbone together with dimensionality reduction and validation-based training optimization to improve computational efficiency and training stability. The dataset used in this study was divided into training (70%), validation (15%), and testing (15%) subsets using stratified sampling to preserve class distributions across all subsets.

Unlike studies focused primarily on developing increasingly complex deep learning architectures, the present work evaluates whether a comparatively lightweight integrated transfer-learning workflow can provide reasonable multiclass diabetic retinopathy classification performance under constrained low-resolution benchmark conditions. The contribution of this study lies not in the introduction of a novel machine learning algorithm, but rather in the integration and evaluation of pretrained feature extraction, dimensionality reduction, and adaptive training strategies within a unified retinal image classification pipeline using a large publicly available dataset.

## 2. Literature Review

Different diabetic retinopathy severity grades were detected from retinal images obtained from the IDRiD dataset using ConvNets, where 80% of the data were assigned to training and the remaining 20% to testing [7]. Ensemble learning models consisted of five CNNs: Resnet50, Inceptionv3, Xception, Dense121, and Dense169. According to the results, the above models succeeded in diagnosing the disease with higher accuracy than the previous methods, especially in the early stages.

An automated DR detection model was proposed using retinal images collected from Melaka Hospital [26]. This model was trained using three neural networks: backpropagation neural network (BNN), deep neural network (DNN), and CNN. According to the results, CNN outperformed BNN and DNN with an accuracy of 93%. Furthermore, target class thresholds were identified using a weighted fuzzy C-means (FCM) algorithm. A new segmentation method, known as EAD-Net, was proposed for feature extraction and pixel-wise label prediction using a ConvNet (sensitivity, specificity, and accuracy of 97% in the *e_ophtha_EX* dataset) [27]. Due to their simpler structure, conventional artificial neural networks (ANNs) generally possess lower processing time and accuracy than their deep counterparts. Image processing is more commonly performed by deep CNNs. The “DeepDR” system was developed using 466,247 retinal images from 121,342 diabetic patients for the detection of diabetic retinopathy across multiple severity grades. In the above system, the area under the receiver operating characteristic curve (AUC) was calculated to be 0.901, 0.941, 0.954, and 0.967 for detecting microaneurysms, cotton wool spots, hard exudates, and hemorrhages, respectively. Also, these figures were 0.943, 0.955, 0.960, and 0.972, for mild, moderate, severe, and proliferative diabetic retinopathy, respectively [28].

A clinical decision-support system (CDSS) (medical decision-support system (MDSS)) was proposed, which comprises four basic steps: imaging, image preprocessing (including localization of retinal structures), feature extraction, and DR classification. A combination of blurred image processing, Hough circle transform, and feature extraction techniques were adopted to improve the performance. The relevant database is available on Kaggle, achieving accuracies of 58% and 85% [29].

An attention module, called CBAM, was proposed to infer the attention map along two dimensions: channel and spatial. These attention maps are then multiplied by the input feature map for (adaptive) feature optimization. CBAM is a lightweight and general module that can be integrated into any CNN architecture. It was tested on three datasets, namely ImageNet-1K, MS COCO, and VOC 2007 (the highest accuracy: 78%) [25].

DR detection was carried out using CNN-based DL algorithms that classify images into two classes [30]. A model with Siamese-like architecture was trained using the TL technique, which accepts binocular (fundus) images as inputs to learn their correlation. The results indicated the positive effect of the binocular design on model efficiency and its superiority over existing monocular models.

CNNs (e.g., AlexNet) were adopted to classify retinal images of the Messidor dataset based on disease severity. This algorithm achieved an accuracy of 96% for healthy and diseased retinal images in steps 1, 2, and 3 [31].

A modified U-Net architecture was proposed for the segmentation of lesions from images to assess disease severity, which defines the targeted region through periodic shuffling and sub-pixel convolution [32]. This model was trained on two publicly available datasets, namely IDRiD and e-ophtha, which obtained a similar accuracy of 99%.

In another study, a deep CNN method was proposed to segment four retinal lesions, which utilizes a collaborative attention mechanism (CAM) architecture and classification to reduce model overfitting. According to the results of the tests performed on the Fundus dataset, the average accuracy was calculated to be 67% [33].

In another study, a relation transformer block (RTB) was proposed to segment retinal lesions (for retinal lesion segmentation) [34]. This model uses self-attention and mutual attention transformers to exploit global dependencies among lesion features and vascular information. To this end, it initially takes small lesion patterns and stores detailed information in a deep network. The IDRiD dataset was utilized, and the proposed method achieved an accuracy of 94%.

Given the considerable importance of the early detection of DR and its positive effect on the general health and quality of life of diabetic patients, it is therefore essential to conduct further research in this field. Studies can help improve individual health, reduce DR treatment costs, and prevent dangerous complications for diabetic patients.

A suitable DR severity grading framework was proposed. The advantages of ML and DL algorithms were combined to establish a robust framework through a trade-off between model processing and classification performance. For this purpose, the fundus images (FIs) were initially preprocessed using CLAHE to highlight the lesions more clearly, then normalized and finally reshaped. A lightweight deep CNN model was then developed to extract the most discriminant features from the processed FIs. The extracted features were standardized to be imported into the extreme learning machine (ELM) algorithm for DR severity-level classification. The model proposed in [35] produced promising results for 34,984 images (dataset 1) and 3662 FIs from the *APTOS 2019* dataset. Not only did it show higher classification performance, but it also significantly reduced parameters, layers, and processing time. This model outperformed existing state-of-the-art (SOTA) models for both datasets and succeeded in the early detection of DR severity with good accuracy. Hence, it helped reduce patients’ vision loss and free doctors’ time.

**Figure 3:**
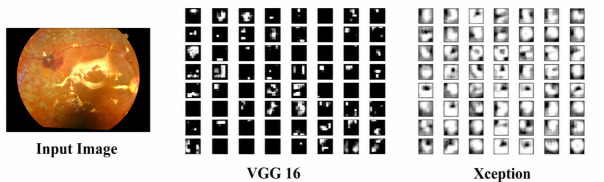
Example feature maps generated from a retinal fundus image using deep convolutional neural networks. The left panel shows the input retinal image, while the middle and right panels show feature maps from the final convolutional layers of the VGG16 and Xception models, respectively [36].

A robust and efficient model was developed for automated DR detection (diagnosis) focused on extracting highly descriptive and discriminative deep features, and subsequently for improving DR detection performance [36]. To get an optimal representation, features were extracted from multiple pretrained CNN models and blended using pooling approaches (multimodal pooling module). These final representations were used to train a deep neural network (DNN) with dropout at the input layer. The proposed model comprises three distinct modules, namely feature extraction, model training, and an evaluation (assessment) module, where the initial representation of retinal images was obtained from pretrained VGG16, NASNet, Xception Net, and Inception ResNetV2. Since each pretrained model required different input image sizes, the given retinal images were reshaped based on the input dimensions accepted by them. For example, the images were reshaped to 224 × 224 × 3 when VGG16 was used. Figure 3 visualizes the feature maps resulting from the final convolution blocks of the VGG16 and Xception models when sending retinal images.

Two distinct pooling approaches (one-dimensional pooling and cross-pooling) were proposed to combine deep multimodal features extracted from VGG16 (fc1 and fc2) and Xception [36]. While 1D pooling is utilized to select locally salient features from each VGG16 region, cross-pooling allows the pooling of salient features obtained by 1D pooling through a global representation of Xception. Each feature element *u_i_* from the output vector *Û* is calculated by one of the following three methods, as in Equations (1)–(4):

(1) $$1D\;Max\;pooling:{\hat{u}}_{i}=max\left(u_{i*2},u_{i*2+1}\right)\;\forall \;i\;\in \;\left\{1,\;2,\;\ldots ,\;d_{2}\right\}.$$

(2) $$1D\;Min\;pooling:{\hat{u}}_{i}=min\left(u_{i*2},u_{i*2+1}\right)\;\forall \;i\;\in \;\left\{1,\;2,\;\ldots ,\;d_{2}\right\}.$$

(3) $$1D\;Average\;pooling:{\hat{u}}_{i}=mean\left(u_{i*2},u_{i*2+1}\right)\;\forall \;i\;\in \;\left\{1,\;2,\;\ldots ,\;d_{2}\right\}.$$

(4) $$1D\;Sum\;pooling:{\hat{u}}_{i}=u_{i*2},u_{i*2+1}\;\forall \;i\;\in \;\left\{1,\;2,\;\ldots ,\;d_{2}\right\}.$$

In another study [32], 3662 images were employed from the Kaggle dataset (70% for training and 30% for testing). To prepare the images for the model, the images were first resized to 224 × 224 × 3.

The proposed method was adopted as an important data augmentation strategy in image enhancement by horizontal and vertical flip, zoom, and channel shift features. The dataset test used batch size 32 and binary cross-entropy as the loss function, ReLU as the activation function, and sigmoid as the activation function in the final layer of the model to reduce model overfitting and increase its accuracy. Adam was also used as an optimizer, and the images were classified into five classes (accuracy = 71.18%).

**Figure 4:**
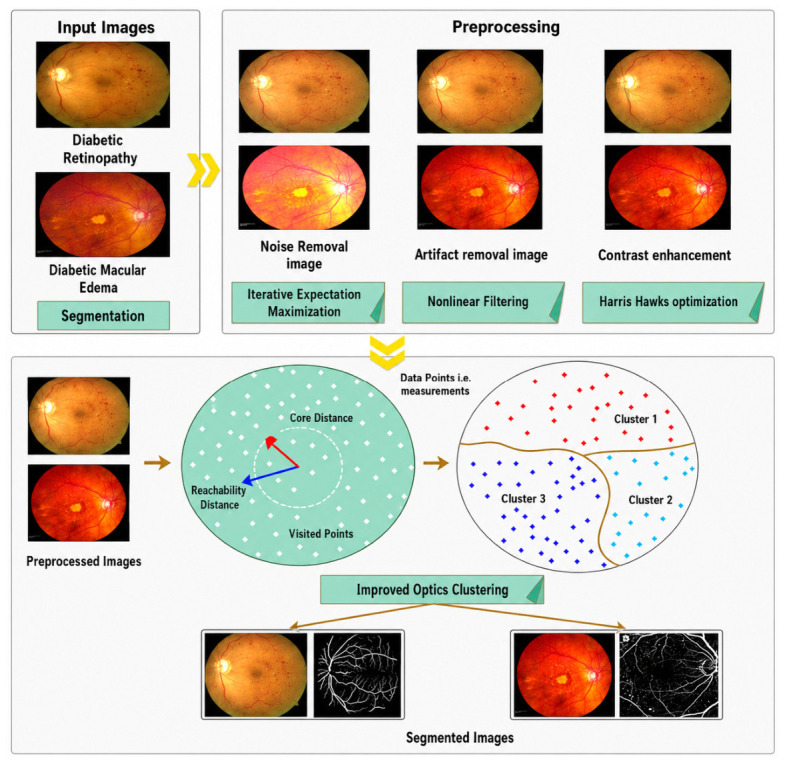
Overview of the diabetic retinopathy detection pipeline proposed in [37]. The pipeline includes retinal image acquisition, preprocessing (noise removal, artifact removal, and contrast enhancement), segmentation, and clustering using an improved OPTICS method to generate segmented retinal images. The arrows indicate the processing workflow between consecutive stages. Different colors represent distinct image-processing steps and clusters identified by the improved OPTICS algorithm. The colored dots denote individual data points used during clustering, while the cluster labels (Clusters 1–3) indicate the corresponding grouped regions. The curved arrows illustrate the reachability and core distances used by the OPTICS clustering algorithm.

In [37], the authors focused on the accurate detection of DR and diabetic macular edema (DME) based on retinal FIs. Disease severity was determined based on the features extracted from the images. Figure 4 depicts an architectural view of the proposed work. The main idea behind OPTICS (Ordering Points to Identify the Clustering Structure) is to extract the clustering structure of a dataset by identifying the density-connected points. This approach creates a density-based representation of the data by creating an ordered list of points called a reachability plot. Each point in the list is associated with a reachability distance, which is a measure of how easy it is to reach that point from other points in the dataset. Therefore, points with similar reachability distances are likely to be in the same cluster.

**Figure 5:**
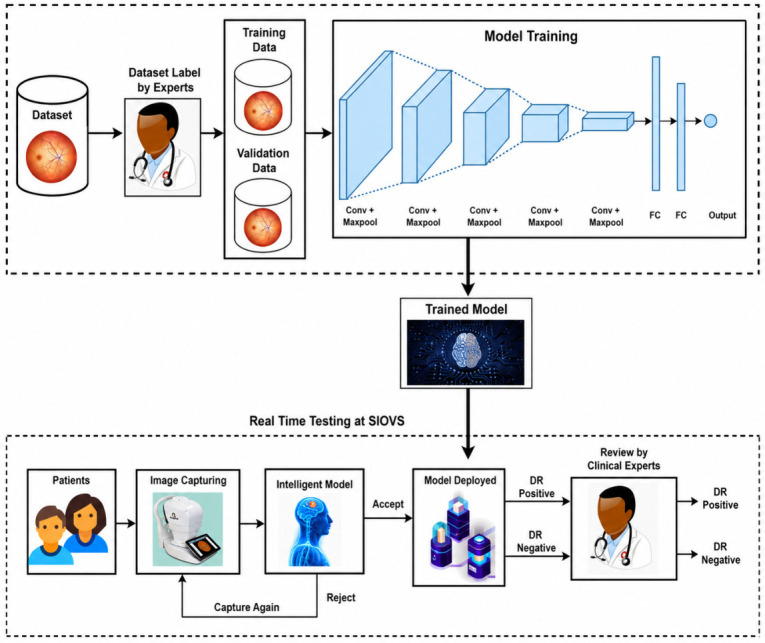
Architecture of a deep-learning-based diabetic retinopathy detection system [38]. The framework includes dataset labeling, model training using retinal images, and real-time deployment where patient images are analyzed by the trained model and verified by clinical experts.

A general DR architecture classification method was presented (Figure 5) [38]. The DR model is composed of two stages: (1) training and validation, and (2) testing. In the first stage, the dataset was compiled from multiple sources, including hospitals and various paid data repositories. The private dataset consists of 57,625 DR images. The experts labeled the dataset as either DR-positive or DR-negative. This dataset was further divided into two subsets: training (80%) and validation (20%). After splitting the dataset, the subsets served as inputs for the CNN-based DR model to train and validate the model. The proposed DR model consists of convolutional and pooling layers.

In convolutional layers, convolutional operations with different filter sizes were performed to extract features from the input dataset. Moreover, the Rectified Linear Unit (ReLU) activation function (AF) was applied to convert linear data to nonlinear data. The pooling layer was activated after the convolutional layer was used. Common types of pooling layers include max pooling, min pooling, average pooling, and sum pooling. Herein, the pooling layer was applied to reduce the size of the activation map without losing important information in the dataset.

Subsequently, the convolutional and pooling layers and the feature map are converted into a vector. The DR model employs multiple fully connected layers (FCLs). Finally, the output was obtained from the DR model in the form of DR-positive and DR-negative results. In the second phase of the study, real-time DR data in the testing dataset were acquired from the patients at the Sindh Institute of Ophthalmology & Visual Sciences (SIOVS), Hyderabad, Pakistan, using image-capturing devices. A real-time testing dataset consisting of images obtained from patients with Type II diabetes (T2D) was created over five weeks. An intelligent model was then used to evaluate the quality of real-time test dataset images. Low-quality images detected by the intelligent model were rejected and then recaptured [38].

In [23], the number of classes was reduced from five to three categories: no DR (grade 0), lower-severity NPDR (grades 1–2), and advanced DR (grades 3–4). The lowest number of images was observed in the advanced DR category (*n* = 488). Therefore, 488 images were randomly selected from the remaining categories (no DR and lower-severity NPDR). These images were transferred to CNN models to extract feature vectors, and the batch size was set to 32. The feature vectors obtained from each model were combined to form a hybrid feature vector to transfer to different classifiers. For further comparison, individual TL models, which only use a DenseNet or ResNet feature vector of 1000 features, were also transferred to the classifiers. The hybrid model proposed in [23] achieved the highest accuracy of 97.8% for binary classification and 89.29% for multiclass classification using the support vector machine (SVM) classifier, indicating its superiority over recent similar binary and multiclass DR detection approaches.

Although prior studies have demonstrated the effectiveness of transfer learning for diabetic retinopathy detection, most approaches primarily focus on selecting or modifying CNN architectures and fine-tuning strategies. In contrast, fewer works explicitly address the combined impact of class balancing, feature-space optimization, and adaptive training within a single, unified screening pipeline. This gap motivates the integrated workflow proposed in this study. In particular, the workflow emphasizes imbalance-aware training and evaluation using class-specific metrics, rather than relying only on overall accuracy.

## 3. Materials and Methods

### 3.1. Overview of the Proposed Method

This study investigates whether combining transfer learning with dimensionality reduction and carefully optimized training strategies can improve the performance and efficiency of machine learning models for diabetic retinopathy (DR) detection. The proposed framework consists of four main stages: image preprocessing, deep feature extraction using a pretrained convolutional neural network, dimensionality reduction in extracted features, and model training with optimized parameters. In this study, the pretrained network used for feature extraction was ResNet50 pretrained on ImageNet.

The central hypothesis is that transfer learning enables effective reuse of visual representations learned from large-scale datasets, while dimensionality reduction and adaptive optimization strategies reduce computational complexity, stabilize training, and improve generalization. The overall workflow of the proposed method is illustrated through these sequential stages and evaluated on a large, publicly available retinal image dataset.

Unlike many prior transfer-learning studies that primarily focus on end-to-end fine-tuning of pretrained convolutional neural networks, the present study evaluates a comparatively lightweight workflow integrating pretrained feature extraction, dimensionality reduction, and adaptive training strategies within a unified retinal image classification pipeline. The goal of this study was not to introduce a novel deep learning architecture, but rather to examine whether coordinated optimization of these workflow components could support multiclass diabetic retinopathy classification under constrained benchmark conditions.

### 3.2. Dataset Description

The dataset used in this study was obtained from the Kaggle repository. It contains 35,126 labeled retinal fundus images categorized into five diabetic retinopathy severity grades (0–4).

Importantly, in this Kaggle version, the images are provided in a downsampled format (28 × 28 pixels, RGB). Because this resolution is not suitable for direct clinical interpretation and is also too small for standard CNN backbones, the images were upsampled during preprocessing to match the input requirements of the transfer-learning model. The resizing procedure was performed solely for compatibility with the pretrained ResNet50 architecture and should not be interpreted as restoration or reconstruction of clinically meaningful retinal anatomy. Image interpolation is a common preprocessing step in transfer-learning pipelines because pretrained convolutional neural networks typically require fixed input dimensions. However, the authors acknowledge that upsampling low-resolution retinal images cannot recover microscopic pathological structures such as microaneurysms or subtle hemorrhages. Therefore, the present study should be considered a proof-of-concept evaluation on a constrained benchmark dataset rather than a clinically deployable diagnostic framework. Upsampling does not recover lost anatomical detail; it only adapts the data format for the network.

All experiments were conducted in Python 3.11 using PyTorch 2.4. Random seeds were fixed across Python, NumPy, and PyTorch, and CUDA non-deterministic operations were controlled where applicable to improve reproducibility.

The dataset contains five labels corresponding to DR grades 0 through 4. Because the dataset is class-imbalanced, all splits were performed using stratified sampling to preserve class proportions. In the present study, the five classes correspond to the standard diabetic retinopathy grading categories commonly used in the literature: Class 0 = No DR, Class 1 = Mild non-proliferative diabetic retinopathy (NPDR), Class 2 = Moderate NPDR, Class 3 = Severe NPDR, and Class 4 = Proliferative diabetic retinopathy (PDR).

### 3.3. Image Preprocessing and Augmentation

Prior to model training, all retinal images were preprocessed to improve visual quality and ensure consistency across samples. Since pretrained transfer-learning backbones such as ResNet50 require fixed higher-dimensional inputs, all retinal images were resized to 256 × 256 pixels using bilinear interpolation. This resizing step was performed to ensure compatibility with the pretrained architecture and to enable computationally efficient transfer learning using ImageNet-based weights. The resizing process does not recover lost retinal anatomical detail from the original low-resolution images but instead adapts the image format for feature extraction within the transfer-learning pipeline. The resolution of 256 × 256 was selected to maintain manageable computational cost while preserving as much spatial information as possible under the constraints of the original dataset.

Additional preprocessing steps included contrast enhancement, optional image sharpening, and random cropping to improve robustness against image variability. To reduce overfitting and increase data diversity, data augmentation techniques such as random horizontal and vertical flips, zooming, and intensity variation were applied during training.

### 3.4. Transfer Learning Backbone and Feature Extraction

Transfer learning was implemented using a ResNet50 convolutional neural network architecture pretrained on the ImageNet dataset. The final fully connected classification layers of the network were removed, and the remaining convolutional layers were used as a feature extractor.

During training, the lower-level convolutional layers were frozen to preserve generic visual features such as edges and textures, while deeper layers were fine-tuned to adapt to retinal image characteristics and DR-related patterns. Feature vectors were extracted after global average pooling of the final convolutional layer, resulting in a 2048-dimensional deep feature representation for each image.

ResNet50 was the only pretrained backbone used in this study in order to isolate the impact of the proposed workflow components rather than architectural variation. The ResNet50 implementation was the standard PyTorch torchvision model, initialized with ImageNet weights. The extracted feature vector was taken after the global average pooling layer, producing a 2048-dimensional embedding per image. The resulting 2048-dimensional deep feature vectors were subsequently used as inputs to the dimensionality reduction stage described in Section 3.5.

### 3.5. Dimensionality Reduction

The deep feature vectors extracted from the ResNet50 backbone after global average pooling have a dimensionality of 2048 features per image. To reduce redundancy and computational complexity, Principal Component Analysis (PCA) was applied to these deep feature embeddings rather than to raw pixel values. PCA projects the extracted feature vectors into a lower-dimensional subspace while preserving the directions of maximum variance.

In this study, the number of principal components was selected to retain approximately 95% of the cumulative variance, ensuring that the most informative features were preserved while significantly reducing dimensionality. This step reduces computational cost, mitigates overfitting, and improves the efficiency of the subsequent classification stage.

### 3.6. Training Strategy and Parameter Optimization

Model training was performed using the Adam optimizer with an initial learning rate of 0.001. To stabilize training and improve convergence, an adaptive learning rate scheduling strategy was employed: the learning rate was reduced by a factor of 0.1 when the validation loss failed to improve for a predefined number of epochs.

The batch size was set to 32, and categorical cross-entropy loss was used as the loss function. Regularization techniques, including data augmentation and early stopping based on validation performance, were applied to reduce overfitting. Hyperparameters (learning rate, scheduler patience, and number of unfrozen layers) were selected iteratively using validation-set performance metrics.

The key hyperparameters and implementation settings used in the proposed framework are summarized in Table 1 to improve reproducibility.

Because the Kaggle diabetic retinopathy dataset exhibits substantial class imbalance, stratified sampling was used when splitting the dataset into training, validation, and testing subsets to preserve class proportions across all sets. In the current implementation, explicit imbalance mitigation strategies such as class-weighted loss functions, focal loss, oversampling, undersampling, or synthetic resampling techniques were not incorporated. Consequently, the observed reduction in sensitivity for intermediate DR stages likely reflects, in part, the combined effects of class imbalance and limited representation of clinically subtle disease categories. Future work will evaluate imbalance-aware learning approaches to improve performance for underrepresented classes.

Hyperparameter selection was performed using validation-based empirical tuning rather than automated search algorithms. Key parameters, including the learning rate, scheduler patience, batch size, and number of unfrozen layers in the transfer-learning backbone, were adjusted iteratively using validation performance to obtain stable convergence behavior during training. Although this approach allowed practical optimization of the training process, it does not guarantee globally optimal parameter selection. More systematic optimization strategies such as grid search, Bayesian optimization, or evolutionary algorithms were not employed in the current implementation but represent important directions for future work to improve reproducibility and potentially enhance model performance.

### 3.7. Training, Validation, and Testing Strategy

**Figure 6:**
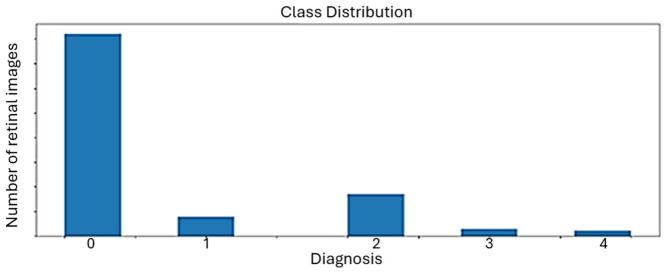
Class distribution of diabetic retinopathy (DR) severity levels in the dataset. The *x*-axis shows DR grades (0–4) and the *y*-axis represents the number of images in each class.

The dataset (n = 35,126) was split into training (70%), validation (15%), and testing (15%) subsets using a stratified split to preserve class proportions. The training set was used to optimize model parameters, the validation set was used for hyperparameter tuning and early stopping, and the test set was used only once for the final evaluation. The class distribution across the three subsets is reported in Figure 6.

## 4. Results and Discussion

### 4.1. Retinal Image Representation

**Figure 7:**
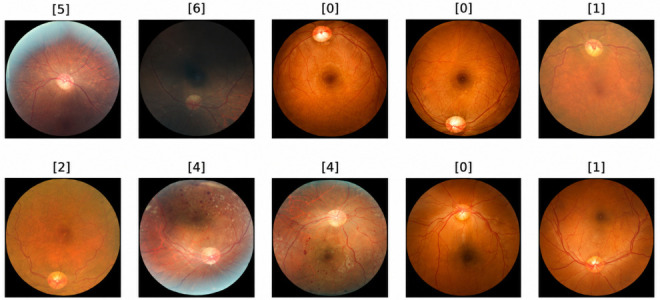
Example retinal fundus images from the training dataset representing different diabetic retinopathy (DR) severity levels (0–4). The images illustrate samples used during preprocessing and data augmentation to improve model training.

This section presents sample images from the training dataset after applying various transformations. As shown in Figure 7, these transformations illustrate both the structural changes associated with diabetic retinopathy in Type 2 diabetes and the preprocessing adjustments applied to the data. This step ensures accurate transformation and prepares the images in a suitable format for model input.

**Figure 8:**
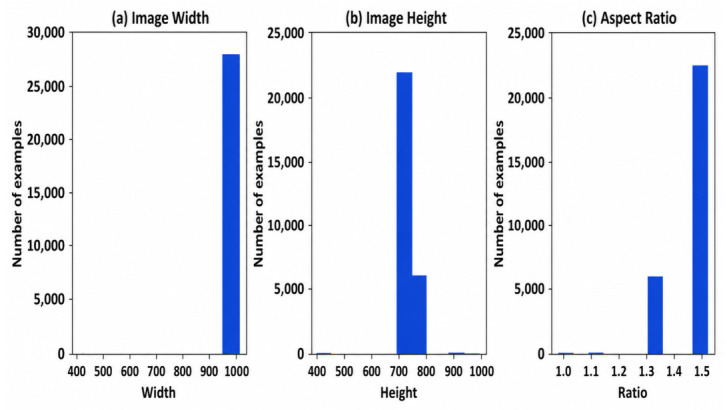
Distribution of retinal image properties in the dataset. The histograms show the distribution of image width (**a**), image height (**b**), and aspect ratio (**c**) across retinal images before resizing and preprocessing.

Each image was accessed using its file path and name, and key attributes were extracted, including height, width, number of channels, and the width-to-height ratio. Figure 8 shows the histograms of these parameters across retinal images before resizing and preprocessing.

The extracted attributes were stored in columns labeled *level*, *height*, *width*, *channels*, and *ratio*. This information was used to analyze image characteristics and guide preprocessing steps such as resizing. In addition, different transformations were applied to the training, validation, and testing sets to increase data diversity and enhance model performance.

### 4.2. Improvement of the Proposed Performance Model

**Figure 9:**
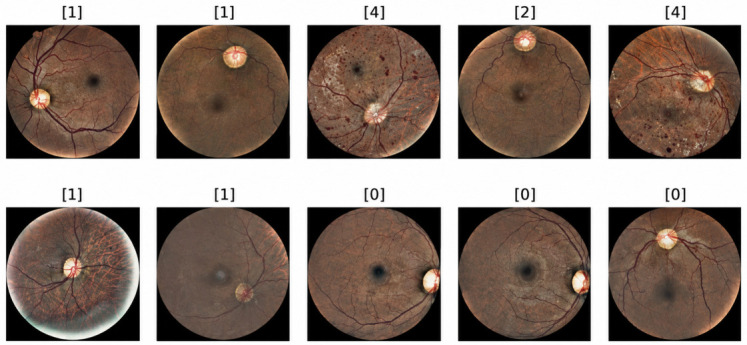
Examples of retinal fundus images after preprocessing and transformation steps applied during model training. The transformations enhance image quality and emphasize retinal structures, supporting improved feature extraction for diabetic retinopathy classification.

As noted earlier, transformations were applied to enhance data diversity and improve model performance during training. Figure 9 illustrates the accuracy of these transformations and the resulting image representations.

**Figure 10:**
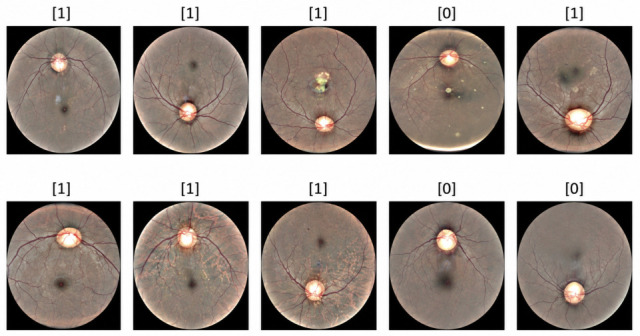
Example retinal fundus images from the testing dataset after preprocessing and transformation steps. These images represent the input data used during the model evaluation stage.

For the testing phase, an *EyeData* class was defined to include the test set (10 initial samples), corresponding image paths, and transformation parameters. A data loader was then implemented to read batches of test data and verify the consistency of transformations and image representations, as shown in Figure 10.

### 4.3. Saving Validation Results in Each Epoch

**Figure 11:**
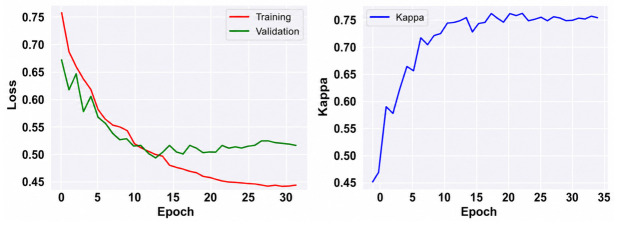
Training dynamics of the proposed model. The left panel shows the training and validation loss values across epochs, illustrating the convergence behavior of the model. The right panel shows the Cohen’s Kappa statistic across epochs, reflecting the agreement between model predictions and ground-truth labels. Error Plot: (**left**) illustrates error dynamics during training and validation. The red and green curves correspond to training and validation errors, respectively, plotted across epochs. Kappa Plot: (**right**) shows the evolution of the Kappa statistic during training. The blue curve represents validation performance, with higher values indicating stronger agreement between predictions and ground truth.

During training, the model parameters were updated through forward and backward propagation, with errors calculated and recorded for each epoch. In the validation phase, the model was evaluated on held-out data to monitor performance and prevent overfitting. Figure 11 presents the training and validation error dynamics, along with the Kappa statistic across epochs.

### 4.4. Evaluation of Final Model Performance

Model performance on validation data was assessed using out-of-fold (OOF) error and the Kappa statistic. The proposed model achieved an OOF error of 0.5004 and a Kappa of 0.7801, reflecting strong agreement with the validation labels. A Kappa value between 0.6 and 0.8 is generally considered good, while values above 0.8 indicate excellent performance. The relatively low OOF error further supports the model’s robustness.

### 4.5. Confusion Matrix

**Figure 12:**
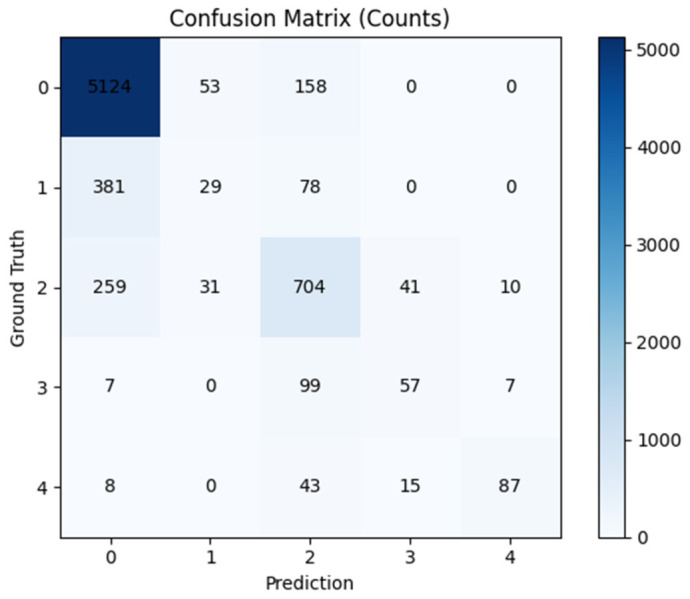
Confusion matrix showing the classification performance of the proposed model on the testing dataset. Rows correspond to the ground-truth diabetic retinopathy (DR) severity classes, and columns correspond to the predicted classes. The values represent the proportion of samples assigned to each class, highlighting correct classifications and misclassifications across DR severity levels (0–4).

A confusion matrix was generated to evaluate classification performance across all classes (Figure 12). The matrix summarizes the number of correctly and incorrectly classified samples, with rows corresponding to actual labels and columns to model predictions.

### 4.6. Report on the Proposed Model Performance

Model performance on the testing data was evaluated using multiple metrics, including accuracy, sensitivity (recall), and F1-score. Table 2 summarizes these results for each class, providing a detailed assessment of classification performance across all severity levels.

Although the proposed model achieves relatively strong overall accuracy, class-wise analysis reveals substantially reduced performance for non-proliferative diabetic retinopathy (NPDR) stages, particularly mild diabetic retinopathy (Class 1), which showed a sensitivity of 0.06 and an F1-score of 0.11. These classes are clinically challenging due to subtle morphological differences and overlapping lesion characteristics, especially in heavily downsampled retinal images where fine pathological structures may not be adequately preserved. The observed performance degradation is further influenced by the imbalanced class distribution in the dataset, where NPDR stages are substantially underrepresented compared with no DR and advanced DR cases. Therefore, overall accuracy alone does not adequately capture clinical utility in imbalanced multiclass medical datasets, and class-specific metrics are necessary for a more informative evaluation. Consequently, the current framework should be interpreted as a proof-of-concept evaluation under constrained data conditions rather than a clinically deployable standalone screening system.

These findings suggest that future model development should prioritize sensitivity improvement for Classes 1–3 (NPDR stages) through imbalance-aware learning strategies, higher-resolution retinal datasets, lesion-focused preprocessing, and more robust feature representation methods.

### 4.7. Comparison with State-of-the-Art Methods

To further assess the performance of the proposed framework, we compared it with representative state-of-the-art DR detection methods reported in the literature. Table 3 summarizes several published approaches, along with their reported datasets, model strategies, and classification performance. The proposed method achieved an overall accuracy of 84% on the Kaggle retinal image dataset, demonstrating reasonable overall classification performance under constrained low-resolution benchmark conditions. However, direct comparison across studies should be interpreted cautiously because prior works differ substantially in dataset size, image resolution, preprocessing pipelines, classification tasks, model architectures, and evaluation methodologies. Several studies reporting higher accuracies were conducted using smaller or private datasets, binary classification settings, ensemble architectures, or higher-resolution retinal images, which are not directly comparable to the constrained benchmark setting used in the present study. Therefore, the comparisons in Table 3 are intended primarily to provide contextual reference rather than definitive performance ranking across studies.

Notably, the approach of Qummar et al. [29] was evaluated on the same Kaggle retinal image dataset containing 35,126 fundus images, allowing a more direct comparison between methods. While Qummar et al. employed an ensemble of five deep convolutional neural networks, the proposed framework uses a single ResNet50 backbone combined with PCA-based feature reduction and adaptive optimization, resulting in a comparatively simpler architecture.

### 4.8. Failure Modes and Misclassification Analysis

Analysis of the confusion matrix reveals that most misclassifications occur between adjacent DR severity levels, particularly among NPDR stages (Classes 1–3). These classes are characterized by subtle and overlapping retinal features, such as early microaneurysms and mild hemorrhages, which are difficult to distinguish even for expert clinicians, especially in heavily downsampled low-resolution retinal images.

In particular, the extremely low sensitivity observed for mild diabetic retinopathy (Class 1) represents a major limitation of the current framework. Early-stage lesions often occupy only a very small portion of the retinal image and may not be adequately preserved in the constrained low-resolution dataset used in this study. In addition, substantial class imbalance further reduced detection performance for underrepresented NPDR categories.

Several factors may explain the reduced performance for Classes 1–3. First, these categories contain subtle lesion patterns, such as microaneurysms, small hemorrhages, and early vascular abnormalities, that may be poorly preserved in low-resolution images. Second, the class distribution is highly imbalanced, limiting the number of representative training examples for NPDR stages. Third, adjacent DR grades share overlapping visual characteristics, making boundary classification difficult even when global image-level features are learned by the model.

Future work should address these limitations through class-weighted loss functions, focal loss, targeted oversampling of underrepresented classes, lesion-aware preprocessing, and training on native high-resolution retinal datasets. In addition, explainability methods such as class activation mapping may help determine whether the model attends to clinically meaningful retinal regions.

The model shows stronger performance in distinguishing fundus images without diabetic retinopathy (Class 0) from more advanced diabetic retinopathy categories (severe NPDR and PDR; Classes 3–4), suggesting greater robustness for more pronounced pathological patterns. However, earlier NPDR stages are substantially more prone to misclassification, reflecting both limited visual separability and dataset imbalance.

From a clinical perspective, underestimation of disease severity in NPDR cases may affect clinical follow-up decisions, although mild NPDR alone does not typically require urgent ophthalmologic referral unless diabetic macular edema is present. Errors involving more advanced disease stages may have greater clinical consequences. Therefore, despite reasonable overall classification performance, the proposed framework should not be considered suitable for standalone clinical screening or autonomous diagnostic decision-making in its current form. Instead, the present study should be interpreted primarily as a proof-of-concept evaluation of an integrated transfer-learning workflow under constrained data conditions.

It should also be noted that many clinically deployed diabetic retinopathy screening systems use referral-based classification rather than detailed multiclass severity grading. In these workflows, referable diabetic retinopathy typically includes moderate NPDR, severe NPDR, PDR, or diabetic macular edema, whereas patients with no DR or mild NPDR without macular edema generally do not require urgent referral. Consequently, the clinical implications of reduced sensitivity for mild NPDR may differ from those associated with misclassification of more advanced disease stages. Future studies should evaluate the proposed framework using clinically relevant referral-based endpoints in addition to multiclass severity grading.

## 5. Discussion

This study evaluated an integrated workflow combining transfer learning, dimensionality reduction, and adaptive training strategies for multiclass retinal image classification under constrained benchmark conditions. The proposed model achieved an overall accuracy of 84%, with sensitivity reaching 97% and an F1-score of 92%. These findings support the feasibility of an integrated transfer-learning workflow for multiclass retinal image classification under constrained benchmark conditions. The primary contribution of this work lies in workflow integration and evaluation rather than the development of a novel deep learning architecture.

In comparison with challenges reported in previous studies such as class imbalance, slower convergence, or limited generalizability, the present framework was designed to improve training stability and efficiency through dimensionality reduction and adaptive optimization strategies. Dimensionality reduction helped decrease computational complexity while preserving critical features, and adaptive learning rate tuning accelerated convergence. These results suggest that careful optimization of training strategies may contribute meaningfully to overall model stability and computational efficiency.

From a clinical perspective, early and accurate detection of DR is vital for preventing vision-threatening complications. Although the model demonstrated relatively strong performance for no DR and advanced diabetic retinopathy categories, sensitivity for mild NPDR remained limited. While mild NPDR alone does not typically constitute referable diabetic retinopathy, improved detection of early disease remains important for comprehensive screening and disease monitoring. Consequently, the present framework should not be interpreted as a clinically validated early-detection system for diabetic retinopathy. By improving efficiency and accuracy, the proposed workflow may provide a basis for future development of computer-assisted retinal image analysis systems following substantial methodological refinement and validation.

Nonetheless, several limitations should be acknowledged. Performance for non-proliferative diabetic retinopathy stages (Classes 1–3) was noticeably lower than for no DR or advanced diabetic retinopathy stages. These stages are clinically critical for early intervention but are inherently difficult to distinguish due to subtle and overlapping retinal features. This limitation is further amplified by the severe class imbalance in the dataset, where NPDR stages are substantially underrepresented. The absence of explicit imbalance-aware learning strategies in the current implementation likely contributed further to the reduced sensitivity observed for non-proliferative diabetic retinopathy stages. Analysis of the confusion matrix further indicates that most misclassifications occur between adjacent DR severity grades, particularly among NPDR stages. These categories are substantially underrepresented in the dataset, which likely contributes to the observed reduction in classification performance. These failure modes reflect the subtle and overlapping retinal features characteristic of early and moderate DR, which are difficult to distinguish even for expert clinicians. Importantly, the model rarely confuses images without diabetic retinopathy with advanced diabetic retinopathy, suggesting that errors are primarily localized to neighboring severity grades rather than gross diagnostic failures. Validation was limited to a single publicly available dataset using a stratified train–validation–test split, without external validation on independent clinical cohorts. Consequently, the generalizability and real-world robustness of the proposed framework remain unproven. Future studies should evaluate the proposed workflow using multi-center clinical datasets and external validation cohorts to assess reproducibility and practical applicability across diverse imaging conditions and patient populations.

As a result, high overall accuracy should be interpreted with caution, and future work will focus on imbalance-aware learning strategies to improve sensitivity for these clinically important classes. From a clinical perspective, the poor sensitivity for mild diabetic retinopathy remains an important limitation because it reduces the model’s ability to reliably detect early retinal abnormalities. However, mild NPDR alone does not typically constitute referable diabetic retinopathy in routine screening programs unless accompanied by diabetic macular edema. In contrast, moderate NPDR, severe NPDR, and PDR generally have greater implications for referral and clinical management. Therefore, although improved detection of mild NPDR remains desirable, the clinical impact of these classification errors may be less severe than errors involving more advanced disease stages. Despite reasonable overall classification performance, the present framework should primarily be viewed as a methodological proof-of-concept rather than a clinically validated screening tool.

Nevertheless, the dataset used in this study represents one of the largest publicly available diabetic retinopathy benchmark datasets, containing more than 35,000 labeled retinal fundus images across five disease severity categories. The use of a large public dataset improves transparency and reproducibility relative to smaller or private cohorts frequently used in prior studies, although external clinical validation remains necessary to establish real-world generalizability.

The extremely low sensitivity observed for Class 1 (mild diabetic retinopathy) reflects the difficulty of detecting subtle early-stage lesions such as microaneurysms and small hemorrhages, which may occupy only a very small portion of the retinal image. In addition, the dataset contains substantially fewer examples of NPDR stages compared to no DR images, which further contributes to the observed performance degradation. From a clinical perspective, this limitation indicates that the proposed model should primarily be viewed as a decision-support tool rather than a standalone diagnostic system. In screening workflows, cases predicted as no DR or mild NPDR should still undergo expert review to ensure that early-stage DR is not overlooked.

An important limitation of the present study is the extremely low spatial resolution of the publicly available dataset used for model development. The original retinal fundus images were provided in a heavily downsampled 28 × 28 RGB format, which substantially limits preservation of clinically relevant anatomical structures. Although the images were resized to 256 × 256 pixels to satisfy the input requirements of the pretrained transfer-learning backbone, this interpolation process does not restore microscopic retinal detail required for reliable clinical diagnosis. Consequently, the proposed framework should primarily be interpreted as a proof-of-concept evaluation of an integrated transfer-learning workflow under constrained data conditions rather than a clinically validated diagnostic system. Future work should evaluate the proposed methodology using native high-resolution retinal fundus datasets to enable clinically meaningful lesion detection and more reliable screening performance.

Overall, this study highlights the feasibility of integrating transfer learning with adaptive training strategies for automated retinal image classification under constrained data conditions. Although the current framework demonstrated reasonable overall classification performance, substantial limitations remain regarding early-stage disease detection, dataset imbalance, and low-resolution image quality. Future work should incorporate imbalance-aware learning methods, external validation cohorts, explainability approaches, and native high-resolution retinal datasets to improve robustness and clinical applicability. It should also be noted that direct statistical comparison with previously published methods is limited by differences in datasets, preprocessing pipelines, and evaluation protocols across studies.

## 6. Conclusions

This study evaluated an integrated workflow combining transfer learning, dimensionality reduction, and adaptive training strategies for multiclass retinal image classification under constrained benchmark conditions. Using retinal fundus images from a large publicly available diabetic retinopathy dataset, the proposed framework achieved an overall accuracy of 84% on the testing dataset. The primary contribution of the present work lies in the evaluation of a unified transfer-learning workflow rather than the development of a novel deep learning architecture or optimization algorithm.

At the class level, performance was strongest for no DR and advanced diabetic retinopathy categories, with maximum class-specific accuracy, sensitivity, and F1-score values of 0.89, 0.97, and 0.92, respectively. However, mild-to-severe non-proliferative diabetic retinopathy stages (Classes 1–3) demonstrated substantially reduced sensitivity and F1-scores, reflecting the difficulty of detecting subtle retinal lesions in low-resolution and class-imbalanced data. Consequently, the proposed framework should primarily be interpreted as a proof-of-concept methodological evaluation rather than a clinically validated screening system.

Future work should prioritize imbalance-aware learning strategies, higher-resolution retinal datasets, lesion-focused feature extraction approaches, and external validation using independent clinical cohorts to improve robustness, generalizability, and sensitivity for early-stage diabetic retinopathy.

## Figures and Tables

**Table 1:** Key Hyperparameters and Implementation Settings.

Parameter	Value/Setting
Backbone network	ResNet50 (ImageNet pretrained)
Input image size	256 × 256 × 3
Deep feature dimension	2048
PCA retained variance	95%
Optimizer	Adam
Initial learning rate	0.001
Learning rate scheduling	Reduce by factor of 0.1 when validation loss plateaus
Batch size	32
Loss function	Categorical cross-entropy
Dataset split	70% training/15% validation/15% testing
Data augmentation	Horizontal/vertical flips, zoom, intensity variation
Early stopping	Based on validation performance
Fine-tuning strategy	Lower ResNet layers frozen; deeper layers fine-tuned

**Table 2:** Performance evaluation results of the model proposed herein on testing data.

Criteria	Accuracy	Sensitivity	F1-Score	Other Criteria
0	0.89	0.97	0.92	5282
1	0.35	0.06	0.11	488
2	0.66	0.68	0.67	1036
3	0.52	0.34	0.41	167
4	0.76	0.57	0.65	153
Accuracy (Precision)			0.84	7126
Macro avg	0.64	0.52	0.55	7126
Weighted avg	0.81	0.84	0.81	712

**Table 3:** Comparison of the proposed method with representative state-of-the-art DR detection approaches (reported results across studies are not directly comparable because of differences in datasets, preprocessing pipelines, image resolution, classification settings, and evaluation methodologies).

Method	Dataset	Model/Strategy	Reported Performance
Rahim et al. [26]	600 color fundus images from 300 patient folders	Fuzzy image processing + Circular Hough Transform + Classifier	Accuracy up to 0.93, specificity up to 1.00, and sensitivity up to 0.9245
Qummar et al. [29]	Kaggle including 35,126 retinal images	Ensemble of five deep CNNs: ResNet50, InceptionV3, Xception, Dense121, and Dense169, for multi-stage DR classification	Accuracy 80.8%, recall 51.5%, specificity 86.72%, precision 63.85%
Butt et al. [35]	APTOS blindness detection dataset from Kaggle; 3662 fundus images	Hybrid transfer-learning feature extraction using GoogleNet + ResNet-18	97.8% accuracy for binary classification and 89.29% for multiclass classification
Bajwa et al. [36]	Private fundus-image dataset, 398 patients	Modified CNN, binary DR classification	Accuracy 93.72%, sensitivity 97.30%, specificity 92.90%
Proposed method	Kaggle, including 35,126 retinal images	ResNet50 + PCA + adaptive optimization	Accuracy = 84%, Sensitivity = 97%, F1-score = 92%

## Data Availability

The datasets analyzed during this study are available in the Kaggle repository: https://www.kaggle.com/datasets/tanlikesmath/diabetic-retinopathy-resized/data (accessed 20 May 2019).

## Abbreviations

CNN	Convolutional Neural Network
DR	Diabetic Retinopathy
ML	Machine Learning
TL	Transfer Learning
AUC	Area Under the Curve
DNN	Deep Neural Network
BNN	Bayesian Neural Network
ELM	Extreme Learning Machine
FCL	Fully Connected Layer
